# Treatment of the sensory and motor components of urges to eat (eating addiction?): a mobile-health pilot study for obesity in young people

**DOI:** 10.1007/s40519-019-00836-z

**Published:** 2020-01-14

**Authors:** Robert A. Pretlow, Carol M. Stock, Leigh Roeger, Stephen Allison

**Affiliations:** 1 eHealth International, 2800 Elliott Ave. #1430, Seattle, WA 98121 USA; 2 Northwest University, 5520 108th Ave. N.E, Kirkland, WA 98033 USA; 3 Flinders University, Registry Road, Bedford Park, SA, 5042 Australia

**Keywords:** Body-focused repetitive behavior, Eating addiction, Food addiction, Sensory addiction, Motor addiction, Behavioral addiction

## Abstract

**Purpose:**

Compelling evidence indicates that an addictive process might contribute to overeating/obesity. We hypothesize that this process consists of two components: (a) a sensory addiction to the taste, texture, and temperature of food, and (b) a motor addiction to the actions of eating (e.g., biting, chewing, crunching, sucking, swallowing). Previously, we reported a mobile health application (mHealth app) obesity intervention addressing the sensory addiction component, based on staged food withdrawal. We propose that the motor addiction component can be treated using cognitive behavioral therapy (CBT)-based strategies for body-focused repetitive behaviors (BRFB), e.g., nail biting, skin picking, and hair pulling.

**Methods:**

The present study tested the effectiveness of CBT-based, BFRB therapies added to the staged withdrawal app. Thirty-five participants, ages 8–20, 51.4% females, mean zBMI 2.17, participated in a 4-month study using the app, followed by a 5-month extension without the app. Using staged withdrawal, participants withdrew from specific, self-identified, “problem” foods until cravings resolved; then from non-specific snacking; and lastly from excessive mealtime amounts. BFRB therapies utilized concurrently included: distractions, competing behaviors, triggers avoidance, relaxation methods, aversion techniques, and distress tolerance.

**Results:**

Latent growth curve analysis determined that mean body weight and zBMI decreased significantly more than in a previous study that used only staged withdrawal (*p* < 0.01). In the 5-month follow-up, participants maintained overall weight loss.

**Conclusions:**

This study provides further preliminary evidence for the acceptability of an addiction model treatment of obesity in youth, and that the addition of CBT-based, BFRB therapies increased the effectiveness of staged food withdrawal.

**Level of evidence:**

Level IV, Evidence obtained from multiple time series analysis with the intervention.

## Introduction

There is increasing interest in whether overeating/obesity stems from an addictive process [1–3], although this notion is controversial [4–6]. The focus in the literature has moved to a substantive debate about the “food addiction” versus the “eating addiction” constructs, which has implications for potential treatments [7]. Food addiction (FA) connotes a substance dependence on ingredients in food, e.g., sugar, and is comparable to drug and alcohol dependence [2]. The FA construct involves addictive eating of certain foods, which are craved, sought out, and eaten in excess [2]. In contrast, Hebebrand et al. [7] proposed the eating addiction (EA) construct primarily due to a dearth of evidence supporting substance dependence on food ingredients. The EA construct involves a behavioral addiction to the act of eating [7]. Schulte et al. [8] challenged the EA construct, arguing that (a) behavioral addictions have no ingested component; (b) addictive-eating potential is not the same for all foods; and (c) even substance dependencies like drugs and alcohol have behavioral components.

While the debate has potential implications for the treatment of pediatric obesity, there have been few studies that have tested the effectiveness of interventions for either FA or EA. Various treatment models have been proposed [9], and Vella and Pai [10] recently provided the first review of treatment strategies that might be useful for FA or EA. Their review suggested further research is needed to test and validate clinical treatments including cognitive behavioral therapy (CBT), which has the potential to improve motivation, emotional regulation, coping strategies, and relapse prevention among patients with addiction.

Our research has focused on CBT-based treatment for EA, which we conceptualize as having sensory and motor components. The treatment was proposed based on a quantitative and qualitative analysis of an interactive website for overweight children and adolescents [1]. This analysis led to the notion of a sensory component of EA. This was conceptualized as involving the pleasurable taste, texture, and temperature of food among young people exposed to the Western food environment, where the sensory aspects of food are engineered to encourage consumption [11].

Our treatment model provides young people with specific CBT-based therapies to address these sensory cues for overconsumption. A previous study (“Study 1”) reported on an intervention that addressed the sensory component of EA, delivered via an iPhone^®^ app [12]. This component has been described as “comfort eating,” for instance using soothing, sweet, creamy foods as self-medication for depression [1, 13]. The sensory addiction component of overeating/obesity is supported by the observation that artificial sweeteners increase cravings for sugar [14]. In a further study [15], the iPhone^®^ app intervention had comparable outcomes, higher retention, and lower cost per patient than usual treatment in a multidisciplinary weight management clinic in a tertiary care setting.

We now propose a motor addiction component of EA. The engineered Western food environment presents a wide variety of foods that are enjoyable to chew, crunch or suck, which might encourage overconsumption amongst susceptible young people. More broadly, the motor component of EA is conceptualized as a behavioral addiction to the mechanical actions of eating either hyperpalatable foods or a wider range of everyday foods that are readily available to the young person. These addictive-like behaviors might include repetitive biting, chewing, crunching, licking, sucking, tongue action, swallowing, and hand-to-mouth motion. These behaviors have previously been described as “nervous eating” [1] and appear to represent displacement of stress, tension, and anxiety.

Stress-induced eating in the laboratory rat might be considered as an animal model for the motor addiction component of overeating. Mildly stressing a rat by placing a padded clamp on the rat’s tail reliably induced licking, gnawing, and eating of standard rat chow in multiple satiated animals [16]. Hyperphagia in rats also was induced by exposure to annoying noise [17]. Similarly, stress-induced eating occurs in humans. Youngsters exposed to a mild stressor ate more snack foods when not hungry than the control group [18].

The treatment of child and adolescent body-focused repetitive behaviors (BFRBs) might provide a useful model for treating the motor component of EA. A number of BFRBs (such as nail biting, skin picking, thumb sucking, cheek biting, hair pulling, and nervous tics) have been likened to behavioral addictions [19, 20], and there are effective CBT-based treatments for BFRBs [21, 22]. Hence, we explored whether these already available CBT-based treatments for ostensible motor addictions could be transferred to the treatment of specific behaviors that might be involved in a motor component of EA—a range of repetitive behaviors including biting, chewing, crunching, licking, sucking, tongue action, swallowing, and hand-to-mouth motion.

The current paper (“Study 2”) reports an intervention for youth overeating/obesity treatment with both (1) a sensory addiction component, and (2) a motor addiction component. The sensory addiction component was addressed using staged food withdrawal, as in Study 1. We hypothesize that the addition of a motor addiction treatment (based on the treatments used for BFRBs) will improve weight loss outcomes for young people in Study 2 compared to Study 1.

## Methods

The study was conducted in a convenience sample of young people who responded to newspaper and radio advertisements in Seattle, Washington, for a “Smartphone app weight loss study”. Children and adolescents were screened for eligibility using an online application followed by a telephone interview. Eligibility criteria included obesity (BMI ≥ 95th percentile), willingness to attend group and phone meetings and weigh foods at meals, and adequate motivation (scores greater than 50 points on a 10-item, 1–10 scale response format questionnaire).

### Procedure

Participants were lent an iPhone^®^ 5S (unless they owned an iPhone) and a wireless Bluetooth body scale (Wahoo Fitness Balance Scale) and a wireless Bluetooth food scale (Escali Corporation), both interfaced to the app. The app was connected to a secure server for data storage and monitoring by the investigators. Participants were informed that they would be compensated a maximum of $200, proportional to completion of requirements of the study (daily weigh-ins, weekly phone meetings and attendance at group meetings).

### The intervention program and mobile health application (mHealth app) implementation

To date, this app intervention has been tested, as proof of concept, in a self-selected cohort of 43 young people with obesity, ages 12–21 (Study 1) [12] and in a cohort of 18 young people, ages 14–18, who were referred to a tertiary obesity clinic [15]. Full details of the intervention and the mHealth application are provided in Study 1 [12]. In brief, addiction-based treatment methods were applied to treat obesity in children and adolescents, delivered as an iPhone^®^ app. The treatment methods were proposed after analysis of an interactive website for youth with obesity that revealed common themes of food cravings, tolerance, withdrawal-like symptoms, and nervous, excessive eating patterns [1]. The app intervention was founded on two addiction-based principles: (a) divide-and-conquer; and (b) staged withdrawal/abstinence. Specifically, the intervention addressed three features of addictive eating behavior: (a) staged withdrawal from participant-identified problem foods; (b) staged withdrawal from snacking between meals; and (c) withdrawal from excessive amounts of foods consumed at meals. The authors reported that a food withdrawal approach was feasible to implement in these samples.

Study 1 addressed only the sensory addiction component of EA. The key difference between the present Study 2 and the previously reported Study 1 was the addition of CBT-based, BFRB methods as treatment for the hypothesized motor addiction component of EA. This consisted of: (a) viewing aversive photos/videos or snapping a rubber band against the wrist to quell eating urges; (b) stress reduction, (e.g., worries management); (c) avoiding triggers (e.g., staying out of the kitchen); (d) relaxation techniques (e.g., deep breathing); (e) competing behaviors (e.g., squeezing hands); (f) distractions (e.g., hobbies), and (h) distress tolerance (e.g., urge surfing). The assumption of urge surfing is that an urge never lasts forever. The individual can therefore “ride out” these urges, by stepping back and observing them but not acting on the impulse [23].

One hundred and seventy-seven iPhone notifications and prompts guided participants through the app program. Participants were prompted daily to log into the app and weigh-in via the Bluetooth scale and answer questions about elimination of problem foods, non-specific snacking, and excessive mealtime amounts. If the participant reported that he/she had eaten a problem food or snacked or eaten more at a meal, the app asked why this had happened and what was the participant’s plan to keep this from happening again. The app queried participants daily about adherence to the sensory and motor addiction treatment methods. A motor addiction method instructed the participant to avoid snacking when arriving home from school by first not snacking for 1 h, then the following day not snacking for 2 h, and 3 h the next day until the participant no longer snacked on arriving home from school. The app asked the participant what distractions he/she had used to avoid snacking that day. Another motor addiction method was the Worry List—a stress reduction feature that prompted participants to journal their current worries and create an action plan for each worry. The app prompted participants weekly to update their worry lists and plans.

In Study 2, the intervention program ran for 17 weeks. Participants were asked to weigh themselves daily via the Bluetooth body scale with weights logged automatically via the app. Weekly 15-min phone meetings were conducted between each participant and their mentor (RP or CS) and four 2–4 h face-to-face group meetings were held. At the end of the 17-week app intervention participants were offered participation in a 20-week follow-up extension study without the app. In the first 10 weeks of the extension participants received weekly phone meetings with a mentor for support and in the second 10 weeks they received one phone meeting half-way through at 5 weeks. Two additional face-to-face meetings were held (at 10 and 20 weeks) during the extension. Participants were asked to continue weighing themselves daily during the 20-week extension, and their weigh-ins were self-reported in the phone meetings and confirmed at the face-to-face meetings.

### Data collection procedures

Participants completed online self-report questionnaires at four face-to-face group meetings held at Northwest University. These meetings were held at baseline (week 0), week 4, week 11 and program completion (week 17). Weight and height measurements were taken using a digital stadiometer (Seca Corp.) by trained senior nursing students.

### Primary and secondary outcomes

The primary outcome was zBMI using the LMS method based on the CDC 2000 growth curves as implemented through the Stata zanthro routine [24]. With respect to secondary outcomes (see Table 1 for variables and coding), we hypothesized that the program would be associated with participants reporting better control over food, reduced frequency of binge eating, and improved self-esteem, satisfaction with life, and happiness.

**Table 1:** Variable definitions and coding

Variable	Description	Coded
Binge eating	At baseline “How many times per week do you binge?” Categorized Yes if ≥ 1 time was reported	0 = no 1 = yes
Control	“How much are you able to control your eating?” Collected at the four face-to-face meetings	1 = not much—5 = the most
Self-esteem	At exit “Rate your self-esteem at the beginning of this study?” “Rate your self-esteem now, at the end of this study?” (1 = really poor to 5 = really good)	Difference between before and after ratings
Happy	At baseline and exit “How happy are you?” (1 = very unhappy to 5 = very happy	Difference between baseline and exit ratings
Satisfied	At baseline and exit “How satisfied are you with your life?” (1 = very dissatisfied to 5 = very satisfied)	Difference between baseline and exit ratings
Self-esteem	“Rate your self-esteem at the beginning of this study?” “Rate your self-esteem now, at the end of this study?” (1 = really poor to 5 = really good)	Difference between before and after ratings
Helpfulness	At exit “How helpful was this app to you for losing weight?”	1 = not at all—5 = the most

### Data analysis

Data processing, descriptive statistics and latent growth curve analyses (LGCAs) were conducted using Stata version 15.1 (StataCorp, College Station, TX, USA). LGCA was used to assess the primary outcome of zBMI weight loss. The LGCAs were performed using zBMI calculated at each of four time points (week 0, 4, 11, and 17) as the observed dependent variables. For participants completing the extension study, two further time points were available at 28 and 38 weeks. Three analysis groups were formed: an Intent to Treat (ITT) group comprising all participants who entered the study; a Per Protocol (PP) group comprising participants who completed the 17-week program; and an extension (Extension) group comprising participants who completed the 20-week Extension study. For the ITT group, missing weight data for participants who did not complete the study was imputed using the last observation carried forward (LOCF) method. Logistic regression was used to identify possible predictors of program completion from a range of independent variables, including gender, age, race, and family type. Changes in the secondary outcomes were assessed using paired-sample *t* tests.

## Results

Sixty-one children and adolescents responded to the initial advertisement. A total of 35 participants met the eligibility criteria and entered the study. Baseline characteristics are shown in Table 2. The mean age was 13.8 years, slightly over half were girls (51.4%) and of Caucasian ethnicity (65.7%). Nearly two-thirds (65.7%) were categorized as obese (≥ 95th to  < 99th BMI percentile) and 34.4% as severely obese (≥ 99th BMI percentile).

**Table 2:** Baseline characteristics

	Male (*n* = 17.49%)	Female (*n* = 18.51%)	Total (*n* = 35)
Age (years)			
10–12	10 (58.8)	7 (38.9)	17 (48.6)
13–15	4 (23.5)	7 (38.9)	11 (31.4)
16–20	3 (17.7)	4 (22.2)	7 (20.0)
Mean (SD)	13.2 (0.83)	14.3 (0.64)	13.8 (0.52)
Race			
Caucasian	13 (76.5)	10 (55.6)	23 (65.7)
Black	0 (0.0)	1 (5.6)	1 (2.9)
Asian	0 (0.0)	3 (16.7)	3 (8.6)
Latino	1 (5.9)	1 (5.6)	2 (5.7)
Other	3 (17.7)	3 (16.7)	6 (17.1)
Family type			
Living with both parents	12 (70.6)	12 (66.7)	24 (68.6)
Single or step family	5 (29.4)	6 (33.3)	11 (31.4)
School absenteeism previous 90 days			
0–2 days	12 (70.6)	9 (50.0)	21 (60.0)
> 2 days	5 (29.4)	9 (50.0)	14 (40.0)
Mean (SD)	2.3 (0.63)	4.9 (1.6)	3.7 (0.91)
BMI [unadjusted, mean (SD)]	31.7 (1.9)	33.1 (1.7)	32.4 (1.2)
BMI (percentile)			
Obese (95th–98th)	10 (58.8)	13 (72.2)	23 (65.7)
Severe obesity (≥ 99th)	7 (41.2)	5 (27.8)	12 (34.3)
Mean BMI percentile (SD)	0.98 (0.00)	0.98 (0.00)	0.98 (0.00)
zBMI(mean, SD)	2.26 (0.1)	2.09 (0.1)	2.17 (0.1)

### Program attrition

Of the 35 participants entering the study, 24 (68.6%) completed the 17-week program. Of those who did not complete the study (*n* = 11), six participants withdrew because of parental issues (e.g., parents unwilling to drive participants to meetings), three did not like the approach, and two lacked the time for the program. Seventeen participants elected to take part in the additional 20-week extension study, and one withdrew after 10 weeks to attend college.

### Program implementation

The majority (22 of 24; 92%) of participants who completed the program were able to identify one or more specific problem foods. Typically, these foods included chocolate, chips (crisps), candy, soda pop, pizza, and ice cream. Of the 22 participants who could identify one or more problem foods, two participants were unable to successfully withdraw (cravings unresolved) from their problem foods.

At baseline all participants (*n* = 33) reported snacking at least daily (frequency mean = 2.54, SE = 0.26) and nearly two-thirds (22 of 33, 60.6%) reported binge eating at least one or more times a week (mean = 1.75, SE = 0.25). Nearly 85% of participants (28 of 33; 84.8%) reported snacking on “whatever food was available”, while only 3 participants (9%) reported snacking on particular foods. In addition, more participants (15 of 32; 46.8%) reported bingeing on whatever food was available, compared to 7 participants (21.8%) who reported bingeing on a particular food. By program completion, 80% of participants (*n* = 24) eliminated snacking entirely while the remainder decreased the frequency. Of the 16 participants completing the program who reported bingeing at baseline, 12 (75%) reported that they no longer binged on food. Nearly all (23 of 24: 96%) participants reduced the weighed amounts of foods consumed at home meals, and on average participants reduced weighed amounts eaten at meals to 52.2% of their starting amounts.

### Bothersome urges to eat

At baseline, 19 (79%) of the 24 participants completing the program reported having bothersome urges to eat that they would like to get rid of. Of these 19 participants, the mean daily frequency of their bothersome urges to eat was 2.45 (SE = 0.46), and they rated how much they wanted to get rid of the urges as a mean = 4.3, SE = 0.25 on a five-point rating scales (1 = not much to 5 = a lot). At program completion, 13 of these 19 participants reported still experiencing bothersome urges to eat, but the mean frequency had significantly reduced (*t*(23) = 4.41, *p* < 0.01) (mean = 0.96, SE = 0.19), compared to baseline (mean = 2.45, SE = 0.46).

Table 3 presents participant ratings of the BFRB treatment methods used in the app, as to how helpful each method was felt by participants for losing weight (1 = not much to 5 = most). Distractions were rated the highest (mean = 4.43), followed by urge surfing (mean = 3.52) and avoiding triggers (mean = 3.52).

**Table 3:** Participant helpfulness ratings of BFRB treatment methods (1 = not much to 5 = most)

Method	Mean	SE
Gross things pictures	1.95	0.27
Avoiding triggers	3.52	0.26
Gross videos	1.52	0.17
Urge surfing	3.52	0.26
Distractions	4.43	0.18
Extreme obesity pictures	1.9	0.28
Rubber band	2.19	0.28
Deep breaths	2.29	0.29
Squeezing hands	2.62	0.27

PP analysis group. Higher scores indicate that the participant rated the method as more helpful to them for losing weight. (*n* = 21) Three participants did not complete these questions

### Weight change and secondary outcomes

**Fig. 1:**
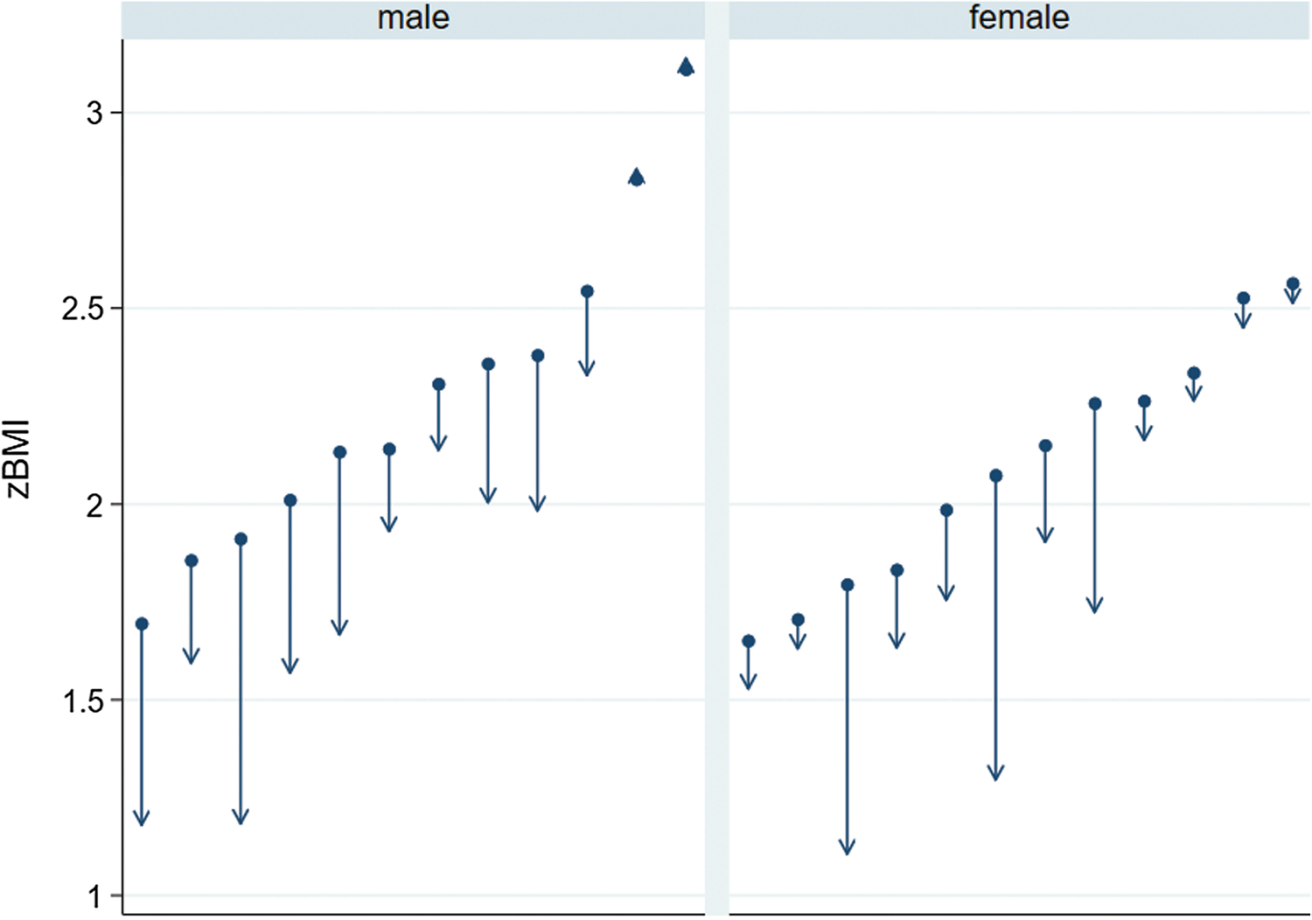
Gender weight loss

Descriptive weight data are presented in Table 4. Both males and females recorded weight loss (measured either by kgs, BMI or zBMI) in the three analysis groups (Intent to Treat, Per Protocol, Extension study). Figure 1 plots initial and program completion for zBMI by gender. The figure highlights the considerable variability between participants both with respect to their initial zBMI weights and weight change.

**Table 4:** Weight change descriptive statistics

	ITT (*n* = 35)		PP (*n* = 24)		Extension (*n* = 16)	
	Baseline	Follow-up*	Baseline	Follow-up^a^	Baseline	Follow-up^b^
kgs						
Male	86.5	83.3	87.7	83.3	74.8	68.6
Female	89.1	85.4	87.3	81.9	78.3	69.5
Total	87.8	84.4	87.5	82.6	76.13	69.0
BMI						
Male	31.7	30.0	32.0	29.7	29.1	26.0
Female	33.1	31.6	33.1	30.8	30.7	26.0
Total	32.4	30.8	32.5	30.2	29.7	26.0
zBMI						
Male	2.26	2.03	2.28	1.97	2.14	1.60
Female	2.09	1.90	2.10	1.83	2.02	1.41
Total	2.17	1.96	2.19	1.90	2.10	1.53

*ITT* intent to treat, *PP* per protocol, *Extension* completed the 20-week extension study

^a^Follow-up 17 weeks

^b^Follow-up 38 weeks

Null (unconditional) LGCAs were estimated using zBMI as the dependent variable for each of the three analysis groups (Table 5). There was a negative zBMI units (week) slope for each of the analysis groups: (ITT: estimate = − 0.013, z = -8.30, *p* < 0.01; PP: estimate = − 0.017, *z* = − 0.9.70, *p* < 0.01; Extension: estimate = − 0.015, *z* = − 0.11.58, *p* < 0.01). This translates to zBMI reductions of 0.22 units over the course of the 17-week program for the ITT group; 0.29 zBMI for the PP group (17 weeks); and 0.57 zBMI over 38 weeks for the extension program.

**Table 5:** LGCAs of zBMI

	Estimate	Std Err	*z*	*p*
Null model (ITT)				
Intercept	2.18	0.70	31.45	< 001
Slope	− 0.013	0.016	− 8.30	< 0.01
Random effect (intercept)	0.160	0.039		
Random effect (slope)	0.014	0.002		
Null model (PP)				
Intercept	2.20	0.085	25.05	< 0.01
Slope	− 0.017	0.002	− 9.71	< 0.01
Random effect (intercept)	0.168	0.049		
Random effect (slope)	0.013	0.002		
Null model (extension)				
Intercept	2.06	0.082	25.84	< 0.01
Slope	− 0.015	0.001	− 9.70	< 0.01
Random effect (intercept)	0.097	0.036		
Random effect (slope)	0.029	0.004		

Participants reported positive improvements across secondary outcomes (PP analysis group). They were better able to control their eating (*t*(23) = 7.02; *p* < 0.01) at program completion (mean = 4.00; standard error [SE] = 0.16), compared to baseline (mean = 2.04; SE = 0.18). Self-esteem improved (*t*(23) = 4.26; *p* < 0.01) from baseline (mean = 2.75; SE = 0.22) to program completion (mea*n* = 3.79; SE = 0.17). Participant happiness ratings were not statistically significantly different (*t*(23) = − 0.15; *p* = 0.88)) between baseline (mean = 3.83; SE = 0.25) and program completion (mean = 3.87; SE = 0.17). Satisfaction with life ratings were also not statistically significantly different (*t*(22) = − 1.0; *p* = 0.33)) between baseline (mean = 3.56; SE = 0.23) and program completion (mean = 3.83; SE = 0.22). Regarding the Worry List feature of the app, 17% of participants (*n* = 24) reported that this feature helped them to avoid overeating when worried/stressed.

The large age range of participants encompassed several developmental stages who might not be expected to respond to the intervention in the same ways. We did not observe any apparent difference in age-related intervention results.

### Comparison with Study 1

The average weight in kilograms (kg) of the 27 participants who completed the 20-week Study 1 at baseline and program completion was: males (113.7:108.7 kg) and females (92.1:91.3 kg). Expressed as zBMI: males (2.5:2.3 zBMI) and females (2.1:2.1 zBMI). In comparison the average weight for the 24 participants who completed the 17-week Study 2 at baseline and completion was: males (87.7:83.3 kg) and females (87.3:81.9 kg). Expressed as zBMI: males (2.28:1.97 zBMI) and females (2.10:1.83 zBMI).

**Fig. 2:**
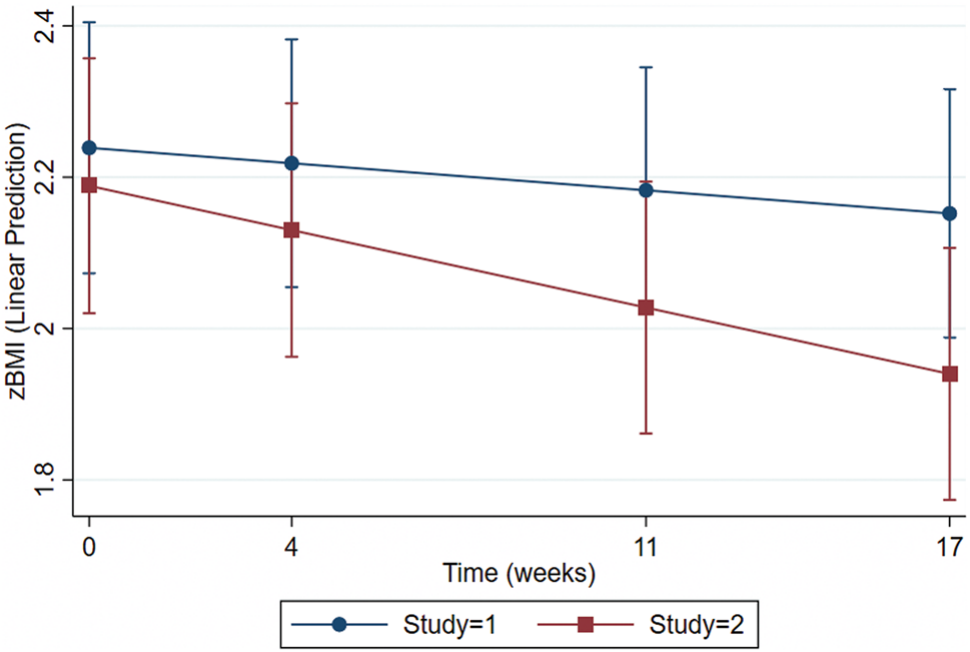
zBMI change, study 1 versus study 2

Figure 2 illustrates that participants in Study 2, on average, achieved statistically significant better weight loss (zBMI estimate = − 0.01, *z* = 4.80, *p* < 0.01) compared to participants in Study 1. This remained statistically significant in a model controlling for gender and age (zBMI estimate = − 0.01, *z* = 4.81, *p* < 0.01).

## Discussion

This study (Study 2) evaluated whether a CBT-based treatment for EA, delivered via an mHealth app platform, would be acceptable to young people and improve their weight loss outcomes. We added a treatment for the hypothesized motor addiction component of EA that was derived from the techniques used for pediatric BFRB. Participants who completed the 17-week program (Per Protocol group) achieved, on average, reductions of 0.29 zBMI. Over the course of treatment, participants also reported reduced binge-eating episodes. While there is not a consensus regarding the threshold for judging the clinical significance of zBMI change, a − 0.25 zBMI change has been found to be associated with clinically significant changes in metabolic and cardiovascular health [25]. Notably, the significantly improved weight loss results achieved in the present study compared with the previous study (Study 1) suggests that the addition of CBT-based, BFRB treatment methods might enhance the addiction-model approach for treatment of obesity in young people. Elimination of snacking by 80% of participants and significant improvement in bothersome urges to eat support the effectiveness of the motor addiction intervention in the app.

Our findings provide partial support for both the FA and EA constructs. Participants in our study reported overeating “problem foods”—highly palatable, craved, sought-after foods, which they were unable to resist or stop eating once started. Cravings for these foods tended to resolve after classic abstinence/withdrawal, derived from substance dependence treatment methods, and this might be interpreted as being consistent with the FA hypothesis. However, participants reported overeating mostly non-specific, everyday foods—whatever was available in the moment—when bingeing and snacking (e.g., a steak in the fridge). Non-specific overeating (whatever food is available) might be interpreted as a hypothetical behavioral addiction (EA), which can be conceptualized as having sensory and motor components with the motor addiction component being predominant.

### Limitations and generalizability

The current pilot study had a relatively small sample size and lacked a control group. A lack of diversity in the sample also limits generalizability to other populations. Future controlled studies in more diverse populations are needed to investigate these preliminary findings further. Nevertheless, the results of the present study on a treatment for the sensory and motor components of EA (Study 2) are promising with a statistically significant decrease in zBMI over the course of the 17-week study, and a statistically significantly greater improvement than Study 1, which treated only the sensory component of EA.

The participant questionnaires used in this study did not include validated measures of either FA or EA, which limits our ability to compare our findings with other studies. We did not use validated measures such as the Yale Food Addiction Scale (YFAS) [26] and the Addiction-like Eating Behavior Scale (AEBS) [27] because their focus is limited to certain foods, while our study had a broader scope, including addictive-like eating of whatever foods were available. The development of a scale that includes the concepts of both: a) addictive-like eating of certain foods, and b) addictive-like eating of whatever foods are available, would be useful for future research.

As this study used neither the YFAS nor the AEBS, there was no direct measure of addiction-like eating behavior variables pre- and post- intervention. Currently, there is no valid and reliable eating addiction scale available; thus, there was no measure of change in eating addiction or change in the motor addiction component (e.g., crunching, chewing, licking) pre- and post- intervention. Nevertheless, our data did show significant elimination of snacking and improvement in bothersome urges to eat, which are consistent with improvement in eating addiction and the motor component.

### What is already known on this subject?

There are no reported treatments for obesity based on models for food addiction or eating addiction, other than two previous articles by the current investigators. Those articles described trials of a withdrawal approach for a hypothesized sensory component of eating addiction. No treatment has yet been described for a hypothesized motor component of eating addiction.

### What does this study add?

This study further supports an intervention for obesity based on the addiction model. It adds a treatment method for a hypothesized motor component of eating addiction to the previously investigated sensory component method and represents a complete treatment for addictive eating behavior. Similarly, this paper notes the need for a scale to evaluate addictive eating behavior that would encompass both sensory and motor components.

## Conclusion

This study provides preliminary evidence for a treatment of EA, which was based on the clinical observation of sensory and motor addiction components of EA associated with youth obesity. The current study findings suggest that the motor addiction component is predominant. The sensory addiction component was treated with staged food withdrawal, whereas the motor addiction component was treated with established CBT-based interventions for pediatric BFRBs. The sensory and motor components were treated separately but concurrently.
